# Helical Twist and Rotational Forces in the Mitotic Spindle

**DOI:** 10.3390/biom9040132

**Published:** 2019-04-01

**Authors:** Iva M. Tolić, Maja Novak, Nenad Pavin

**Affiliations:** 1 Division of Molecular Biology, Ruđer Bošković Institute, Bijenička cesta 54, 10000 Zagreb, Croatia; majanovak505@gmail.com; 2 Department of Physics, Faculty of Science, University of Zagreb, Bijenička cesta 32, 10000 Zagreb, Croatia

**Keywords:** mitotic spindle, forces, microtubules, motor proteins, rotational forces, torque

## Abstract

The mitotic spindle segregates chromosomes into two daughter cells during cell division. This process relies on the precise regulation of forces acting on chromosomes as the cell progresses through mitosis. The forces in the spindle are difficult to directly measure using the available experimental techniques. Here, we review the ideas and recent advances of how forces can be determined from the spindle shape. By using these approaches, it has been shown that tension and compression coexist along a single kinetochore fiber, which are balanced by a bridging fiber between sister kinetochore fibers. An extension of this approach to three dimensions revealed that microtubule bundles have rich shapes, and extend not simply like meridians on the Earth’s surface but, rather, twisted in a helical manner. Such complex shapes are due to rotational forces, which, in addition to linear forces, act in the spindle and may be generated by motor proteins such as kinesin-5. These findings open new questions for future studies, to understand the mechanisms of rotational forces and reveal their biological roles in cells.

## 1. Forces in the Mitotic Spindle

**Figure 1:**
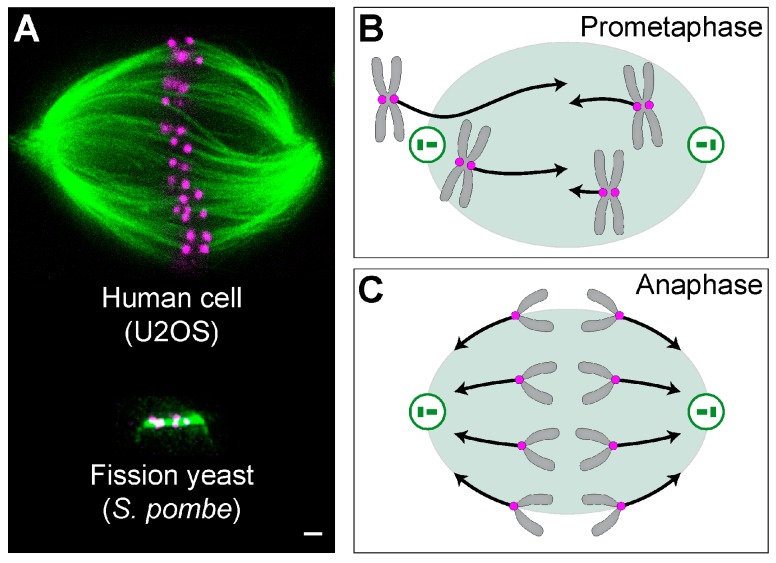
Mitotic spindles and forces acting on chromosomes. (**A**) A mitotic spindle of a human cell (top) and yeast (bottom). Top: a superresolution image obtained by stimulated emission depletion (STED) microscopy (single *z*-plane) of a metaphase spindle in a live U2OS cell expressing CENP-A-GFP (magenta), with microtubules labeled with SiR-tubulin (green). The image is reproduced with permission from [10]. Bottom: image of a spindle from the fission yeast *Schizosaccharomyces pombe*, expressing tubulin labeled with GFP (green), and the kinetochore protein Ndc80p labeled with tdTomato (magenta) [11]. Scale bar is 1 µm for both images. Scheme of forces (black arrows) acting on chromosomes (gray) in (**B**) prometaphase and (**C**) anaphase. Magenta circles represent kinetochores, green circles are centrosomes with centrioles inside, and the green shaded area marks the spindle region.

The mitotic spindle is a fascinating micromachine based on microtubules and their associated proteins, which divide duplicated chromosomes between two nascent daughter cells (Figure 1A). Forces exist in the spindle throughout mitosis and are crucial for spindle functioning in each phase [1,2,3,4,5,6]. In prometaphase, forces are orchestrated in such a way that leads to chromosome congression from a random position to the equatorial plane of the spindle [4] (Figure 1B). In metaphase, forces are balanced to keep the chromosomes under tension at the metaphase plate, which contributes to silencing of the spindle assembly checkpoint [7]. In contrast to prometaphase, the forces in anaphase act towards the spindle poles to move sister chromatids apart [3] (Figure 1C). Thus, precise regulation of the direction and magnitude of forces in space and time is crucial for proper chromosome segregation.

These forces are generated by spindle microtubules through their contact with chromosomes and with other microtubules. The forces arise from active processes of motor proteins and microtubule polymerization and depolymerization [8,9]. The length of the microtubules enables them to establish spatial organization of the spindle and to create bundles that connect chromosomes to spindle poles, whereas their stiffness allows them to withstand and generate forces.

Though it is difficult to directly measure forces by the available experimental techniques, several studies were able to tackle this challenge. In examples of pioneering work, forces that the spindle exerts on a single chromosome in anaphase have been measured using a flexible glass needle [12,13]. More recently, a spring-like force exerted by astral microtubules has been measured by using magnetic tweezers to displace the spindle [14].

## 2. From Forces to Shapes and Back

Forces in the spindle are important not only to drive chromosome movements, but also to generate the shape of the spindle. Spindles come in different shapes depending on the species and cell type. For example, the spindle in animal and human somatic cells has a complex architecture whereby numerous microtubule bundles are organized in a recognizable spindle-shaped structure [15] (Figure 1A, top). By contrast, spindles in lower eukaryotes, such as yeasts, consist of a single microtubule bundle connecting the spindle poles [16] (Figure 1A, bottom).

**Figure 2:**
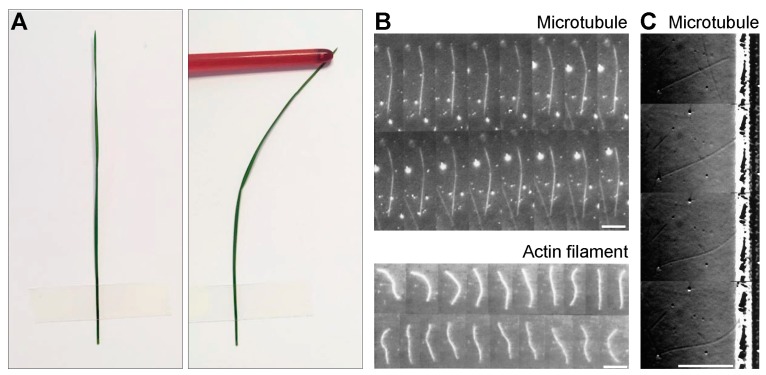
From forces to shapes and back. (**A**) A macroscopic example of a plant stem illustrates the fact that microtubules are intrinsically straight, but can curve under forces. (**B**) Time-lapse images of a microtubule (top; images are separated by 10 s) and an actin filament (bottom; images are separated by 1.5 s) undergoing thermal fluctuations in curvature. The scale bars represent 10 µm. The curvature fluctuations of the microtubule are small due to its high rigidity. The images are reproduced with permission from [17]. (**C**) Images of a buckling microtubule encountering a wall (separated by 1 min). Scale bar, 10 µm. The images are reproduced with permission from [18].

To understand the forces in the spindle, it is important to consider the mechanical properties of microtubules. Microtubules are long and thin flexible polymers which are intrinsically straight, but they can curve under forces [8], which can be illustrated by a macroscopic example of a plant stem (Figure 2A). The shape of the microtubule is more curved for higher forces, where its flexural rigidity describes this relationship (Section 2.1). Due to the simple relationship between the shape, force, and flexural rigidity, knowing two of these quantities is sufficient for determining the third.

This reasoning was used in an elegant work where flexural rigidity of microtubules was measured by resolving the bending of single microtubules due to thermal forces [17] (Figure 2B, top). The mean flexural rigidity of taxol-stabilized microtubules was determined to be 2.2 × 10^-23^ Nm^2^. This flexural rigidity implies that microtubules have a persistence length of more than 5 mm, which means that the observed deformations of microtubules within a cell are caused by forces other than thermal forces. For comparison, deformations of actin filaments induced by thermal forces are more pronounced, yielding a flexural rigidity that is two orders of magnitude smaller than for microtubules [17] (Figure 2B, bottom).

By using a similar approach, pushing forces exerted by growing microtubules against a barrier were determined from the measured microtubule shapes and the known flexural rigidity [18] (Figure 2C). The results show that microtubule growth velocity decreases with an increasing force. The velocity decreased 6 times when the force was increased from 0 to 3-4 pN. Taken together, these in vitro experiments illustrate how shapes of single microtubules can be used to determine their mechanical properties and the forces they can generate.

### 2.1. Deformation of a Slender Elastic Rod

A microtubule can be described as an elastic rod, which is straight and deforms when linear or rotational forces (torques) act on it. Rotational forces can have a component parallel to the rod axis, which induces twisting of the rod, and a perpendicular component, which bends the rod. Linear forces can be, for example, pushing on one end of the rod, which results in bending (Figure 2A).

For a given torque, M, the shape of the microtubule is described using the static Kirchhoff equation [19]:

$$\kappa t\;\times \frac{dt}{ds}+\tau \frac{d\phi }{ds}t=-M.$$

Here, the contour of the microtubule is described by a contour length, s, and a vector pointing from the origin of the coordinate system to a point on the microtubule contour, r(s). The normalized tangent vector is calculated as t=dr/ds. The torsion angle, ϕ(s), describes the orientation of the cross-section along the length of the microtubule. The constants κ and τ denote flexural and torsional rigidity of the microtubule, respectively. To calculate the shape, it is necessary to know the torque. For example, in a simple case when force $F_{0}$ and torque $M_{0}$ act on a microtubule end, the torque in Equation (1) can be calculated as $M\left(s\right)=-r\times F_{0}+M_{0}$ [10].

## 3. Linear and Rotational Forces That Shape the Spindle

Similarly to the case of single microtubules, forces acting in the spindle can be determined from the spindle shape. To make a link between forces and spindle shape, a theoretical model describing interpolar microtubules was introduced [20]. This model includes linear and rotational forces (torques) acting at the spindle poles, which can buckle and bend the microtubules, respectively. The model also describes sliding forces in the overlap region of interpolar microtubules, and forces acting at the plus ends of microtubules that are in contact with chromosome arms. According to this model, interpolar bundles are under compression. Even though this model describes only interpolar bundles, it provides an explanation for the shapes of *Drosophila* embryo spindles.

**Figure 3:**
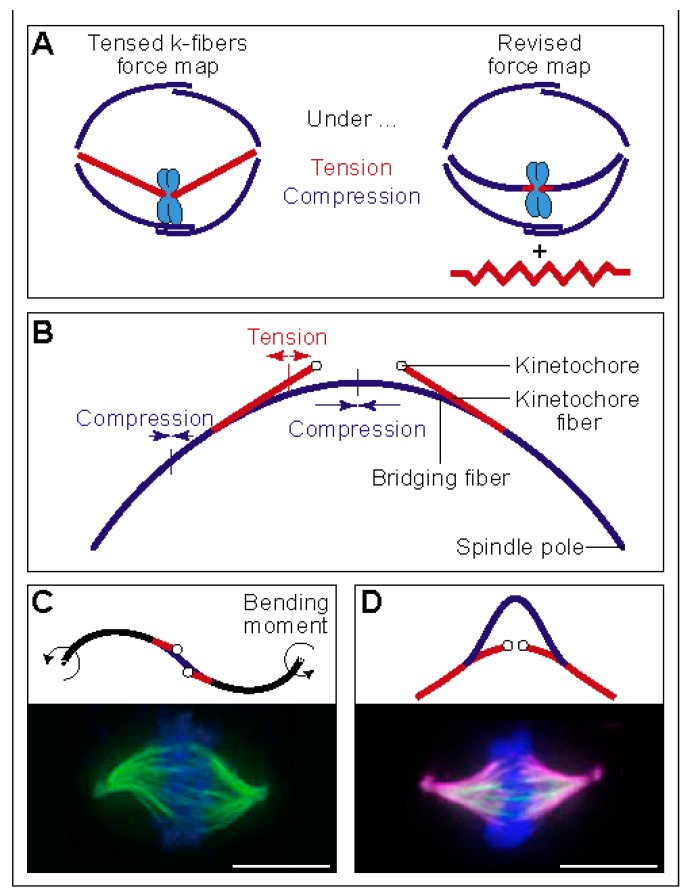
Linear and rotational forces that shape the spindle. (**A**) A classic force map of the spindle, in which kinetochore fibers are under tension and overlap microtubules under compression (left), and a revised force map, where tension and compression coexist along the kinetochore fiber (right). The red element next to the spindle represents a possible non-microtubule component under tension. The image is reproduced with permission from [25]. (**B**) Force balance based on a theoretical model that includes a bridging fiber as a link between sister kinetochore fibers. The compression in the bridging fiber balances the tension on kinetochores and the compression at the spindle pole. Thus, the bridging fiber allows coexistence of tension and compression (red and blue segments, respectively) within an individual kinetochore fiber. The image is reproduced with permission from [26]. (**C**) Top: Predicted shape of a microtubule bundle, resembling the letter “S”, calculated here by using the model from [26] with the parameters: L=11.5 μm, $x_{j}=4.3\;\mu m$, $d_{k}=1\;\mu m$, $\theta_{0}=64{}^{\circ}$, $n_{k}=17$, $n_{b}=14$, $\kappa_{0}=30\;\mathrm{pN}\cdot {\mu m}^{2}$, $F_{k}=280\;\mathrm{pN}$, $M_{0}=403\;\mathrm{pN}\cdot \mu m$, and force at the pole in the y -direction, $F_{0}=83\;\mathrm{pN}$. Bending is present in the black segments. Bottom: S-shaped spindle in a lung cancer cell line. Microtubules are shown in green and DNA in blue. The image is reproduced with permission from [33]. (**D**) Predicted shape of a microtubule bundle that looks like a curved letter “A”, calculated here by using the model from [26] with parameters: L=11.1 μm, $x_{j}=2.7\;\mu m$, $d_{k}=1\;\mu m$, $n_{k}=17$, $n_{b}=8$, $\kappa_{0}=30\;\mathrm{pN}\cdot {\mu m}^{2}$, $F_{0}=-58\;pN$, $F_{k}=280\;\mathrm{pN}$, $M_{0}=0$. Bottom: A-shaped spindle in a HeLa cell overexpressing HSET (kinesin-14). Microtubules are shown in magenta, HSET in green, and DNA in blue. The image is reproduced with permission from [34]. Scale bars, 10 µm.

Similar to interpolar microtubules, outermost kinetochore fibers are also curved, which is indicative of compressive forces. However, the plus end of the kinetochore fiber is under tension, which was deduced based on the observed stretch of the elastic centromeric chromatin that connects sister kinetochores [21,22,23,24]. These findings create a paradox: how can compression turn into tension along the length of a kinetochore fiber between the spindle pole and the kinetochore [25] (Figure 3A). As compression cannot turn into tension without additional forces acting on the kinetochore fiber, a hypothesis was put forward that the spindle matrix balances the forces in the spindle [25].

An alternative solution to the paradox of coexisting tension and compression along a single kinetochore fiber has been provided in our recent study [26]. The key to this solution is a bundle of microtubules termed “bridging fiber”, which links sister kinetochore fibers. The compression in the bridging fiber balances the tension between sister kinetochores, thereby allowing the kinetochore fiber to be under compression in the region close to the pole and under tension close to the kinetochore (Figure 3B). Thus, the paradox can be resolved by taking into account the forces in the bridging fiber, or by a combination of these forces and those generated by the spindle matrix.

The existence of bridging fibers was shown by confocal microscopy, and laser cutting experiments demonstrated lateral interactions between bridging and kinetochore fibers in metaphase [26,27,28,29]. When the bridging fiber was perturbed, for example, by laser cutting of microtubules close to the kinetochore or by silencing of the microtubule crosslinker PRC1 (protein regulator of cytokinesis 1), sister kinetochores moved closer to each other, suggesting that the bridging fiber balances the tension between them [26,30,31]. In anaphase, sliding of the microtubules within the bridging fiber pushes sister kinetochore fibers apart to segregate chromosomes [32].

The balance of forces depicted in Figure 3B is explained by a theoretical model, which we developed to deduce the forces from the measured spindle shape and the known flexural rigidity of microtubules [26]. This model includes linear and rotational forces acting at the spindle pole and tension at the kinetochore. The model predicts a compressive force at the spindle pole of approximately 30 pN, resulting in the compression and buckling of the bridging fiber. The predicted tension at the kinetochore is 50–500 pN, which propagates along the segment of the kinetochore fiber up to 1 µm from the kinetochore, where the kinetochore fiber meets the bridging fiber [26] (Figure 3B). The compressive forces at the spindle pole generate the curved shape of the outermost kinetochore fibers resembling the letter “C”.

Interestingly, the model predicts dramatic changes in the shapes of microtubule bundles when the direction and type of force is changed. If rotational forces act at the spindle pole instead of linear forces, shapes resembling the letter “S” appear (Figure 3C, top). Such shapes can be observed in the central part of the spindle (Figure 1A, top), and whole spindles can attain an S-shape under certain conditions, such as in a lung cancer cell line [33] (Figure 3C, bottom). In another example, if the entire kinetochore fiber is under tension, shapes that look like a curved letter “A” are obtained (Figure 3D, top). Also here, spindles with such shapes have been observed under particular conditions, for example, if HSET (kinesin-14) is overexpressed [34] (Figure 3D, bottom). How these unusual spindle shapes are generated is not known, and it will be interesting to quantitatively compare the shapes from the model and experiments, in order to understand the forces acting in these spindles.

## 4. Twisting Moment in the Spindle Leads to its Chirality

**Figure 4:**
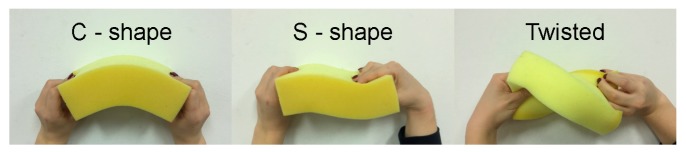
A macroscopic example in which a sponge is deformed by hand, illustrating the relationship between the rotational force and shape. The sponge can be bent in two different ways (left and middle) and twisted (right).

Rotational forces can deform an elastic object in different ways, where the shape of the object provides information about the force direction. The relationship between the rotational force and shape can be illustrated by a macroscopic example in which we deform a sponge by hand (Figure 4). If the rotational force is perpendicular to the long axis of the sponge, which is termed the bending moment, the sponge can attain the shape of a letter “C” or “S”. The C- and S-shapes appear when the bending moments at the two ends of the sponge act in the opposite or in the same direction, respectively (Figure 4, left and middle). However, if the rotational force is parallel to the sponge axis, which is termed twisting moment, the sponge becomes twisted (Figure 4, right).

Indeed, our calculations confirm the hints obtained from the macroscopic example with the sponge. The two-dimensional shapes resembling the letter “S” appear in the theoretical model when rotational forces act perpendicular to the pole-to-pole axis (Figure 3C). However, rotational forces in any other direction lead to three-dimensional shapes with a helical twist [10]. Thus, our model predicts helical shapes of microtubule bundles in the spindle when a component of the rotational force parallel to the pole-to-pole axis exists.

**Figure 5:**
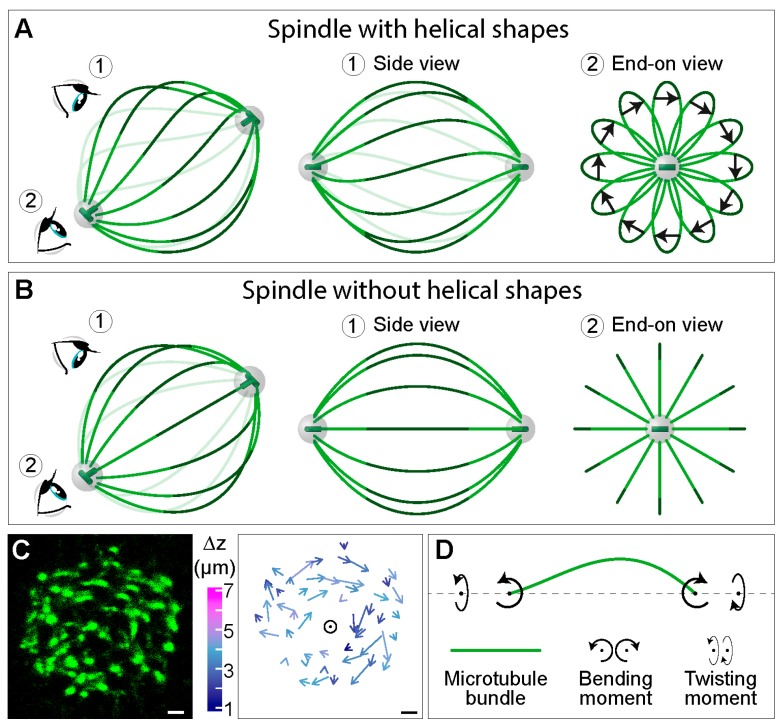
Twisting moment in the spindle leads to its chirality. Simplified scheme of a spindle (**A**) with and (**B**) without helical shapes of microtubule bundles. In each panel, a view of the spindle from an arbitrary angle is shown at the left, together with eye signs marking the view angle for the side view (1) and the end-on view (2), which are shown in the middle and on the right, respectively. Green lines depict interpolar microtubule bundles, the central part of which is marked in a darker shade. The arrows in (A) connect the end points of the central part of each bundle, following the bundle in the direction towards the observer. Grey spheres represent centrosomes with centrioles (green) inside. (**C**) Left, cross section of a spindle in a HeLa cell expressing PRC1-GFP (green), which marks the central part of interpolar bundles, i.e., bridging fibers [30]. Right, arrows connecting starting and ending points of PRC1-GFP bundles traced in the direction towards the observer. Longer arrows roughly correspond to larger twist around the spindle axis (circle) and colors show *z*-distance between starting and ending points (see color bar to the left). Scale bars, 1 μm. The images are reproduced with permission from [10]. (**D**) Scheme of an interpolar microtubule bundle (green) illustrating a physical explanation of the bundle shape, where twisting and bending moments (curved arrows) acting on the bundle generate the observed chiral shapes.

A usual side view of the spindle provides abundant information about the complex shapes of microtubule bundles (Figure 1A, top), but it is not sufficient to determine whether these shapes lie in a single plane or extend in three dimensions as helical shapes, for example. Helical shapes can be better visualized by looking at the spindles along the pole-to-pole axis (end-on view in Figure 5A). In this view, interpolar microtubule bundles look like petals of a flower, whereas in a spindle without helical shapes, the bundles look like straight lines arranged as an aster (compare end-on view in Figure 5A,B). The difference between two cases is most pronounced in the central part of the bundles (dark green segments in Figure 5A,B), where bundles have an arc-like shape in contrast to straight lines in the spindle with and without helical bundles, respectively. The handedness of the bundles can be determined by connecting the end points of each arc by an arrow, following the bundle in the direction towards the observer (Figure 5A). If an arrow rotates clockwise with respect to the spindle pole (as in the end-on view in Figure 5A), the handedness of the bundle is left. Such bundle and its mirror image are not superimposable, thus, the bundle is chiral. If the bundles in an individual spindle predominantly show the same handedness, the spindle is chiral (Figure 5A).

To explore the three-dimensional contours of microtubule bundles in the spindle, we looked at the spindles along the pole-to-pole axis. Microtubule bundles, which appear as spots in a cross-section of the spindle, were tracked through the *z*-stacks [10] (Figure 5C, left). Surprisingly, we found that the arrows connecting the end points of the central part of each bundle rotate clockwise, implying that bundles follow a left-handed helical path along the pole-to-pole axis [10] (Figure 5C, right). Thus, the spindle is a chiral object.

The experimentally measured three-dimensional shapes of microtubule bundles can be used to deduce linear and rotational forces in the spindle by comparison with a theoretical model [10]. The model predicts that a twisting moment of roughly −10 pN·µm can explain the measured twist of the bundles, which turn by about −2 deg·µm^−1^ around the spindle axis. To explain the overall shape of the bundles, the model predicts a bending moment of 140 pN·µm together with the twisting moment. Thus, twisting moment, in addition to bending moment and linear forces, exists in the spindle and determines its chiral architecture (Figure 5D).

## 5. Rotational Forces Generated by Motor Proteins

How are the helical shapes of interpolar microtubule bundles generated? The left-handed twist of the bundles disappears when kinesin-5 (Kif11/Eg5) motor is inactivated during metaphase, suggesting that this motor is important for the maintenance of helical shapes [10]. This motor is known to be crucial for spindle assembly [35,36], yet dispensable for spindle maintenance in many human cell lines [37]. The role of this motor in the generation of helical shapes will be an interesting topic for future studies.

The function of the motors is typically described in terms of linear forces, which may be pulling or pushing forces depending on the direction. Interestingly, motors also exert rotational forces on the microtubule. In vitro studies have shown that axonemal dynein [38], kinesin-14 (Ncd) [39], kinesin-5 (Eg5) [40], kinesin-8 (Kip3) [41], and cytoplasmic dynein [42] can generate rotational forces on the microtubule by stepping sideways while walking along the microtubule. For example, Kip3 motors switch microtubule protofilaments with a bias toward the left, and molecular modeling suggests that this bias is due to the asymmetric geometry of the motor neck linker complex [41].

Unlike these motors, kinesin-1 walks along the microtubule protofilament as it steps from one tubulin dimer to the next, without stepping sideways. Yet, a recent study has shown that kinesin-1 can generate rotational forces on the microtubule by rotating around the axis perpendicular to the microtubule [43]. The rotational forces generated in this manner can reach 1.65 pN°µm. If the motors required for spindle chirality generate similar rotational forces, 10–100 motors per microtubule bundle may generate the helical shapes based on the estimated bending and twisting moments in the bundle.

We speculate that motors can generate rotational forces at two locations in the spindle: in the overlap zone of antiparallel microtubules and at the spindle pole. In the overlap zone, motors may turn antiparallel microtubules around each other while sliding them, which may lead to helical twisting of the bundle. At the pole, motors attached to the pole may rotate microtubules while walking along them towards the spindle equator. New experiments and new theoretical models are necessary to understand how rotational forces are generated and balanced in the spindle.

## 6. Conclusions

Determination of forces in the spindle by using the shapes of microtubule bundles has been a useful approach to understand force balance in the spindle. By using this approach, it has been shown that both tension and compression are present along a single kinetochore fiber, which are balanced by a bridging fiber connecting sister kinetochore fibers. A generalization of this approach to three dimensions revealed that microtubule bundles are twisted in a helical manner, due to rotational forces, which may be generated by motor proteins such as kinesin-5. These findings open an exciting new area of research, where the mechanisms and the biological roles of rotational forces in cells will be revealed.
